# Nursing Students' Experiences of Peer Support During Clinical Practice: A Qualitative Meta‐Synthesis

**DOI:** 10.1002/nop2.70693

**Published:** 2026-07-17

**Authors:** Lianhua Xu, Yunhua Hou, Wei Xiang

**Affiliations:** ^1^ Department of Nursing Wuxi Higher Health Vocational Technology School Wuxi China

**Keywords:** meta‐synthesis, nursing students, peer support, qualitative research

## Abstract

**Aim:**

To synthesise nursing students' experiences of peer support during clinical practice.

**Design:**

A qualitative meta‐synthesis.

**Methods:**

We searched the Cochrane Library, Embase, PubMed, Web of Science, CNKI, Wanfang and VIP databases for qualitative studies exploring nursing students' experiences of peer support, up to 11 September 2024. The Joanna Briggs Institute Qualitative Appraisal and Review Instrument was used to assess methodological quality, and findings were synthesised using a thematic synthesis approach.

**Results:**

Ten studies were included. From 35 extracted findings, eight categories were developed and further synthesised into two overarching themes: (1) benefits of peer support for nursing students, and (2) challenges associated with peer support experiences.

**Conclusion:**

Peer support in clinical practice offers meaningful benefits—including enhanced confidence, emotional support and skill development—but also presents challenges related to mentor competence, interpersonal dynamics and reduced hands‐on practice. Structured programme design and mentor preparation are essential to maximise benefits while mitigating risks.

**Implications for Nursing Practice:**

The findings suggest that peer support should be formally integrated into clinical placement designs to enhance student learning and well‐being. In nursing education, structured peer mentoring programmes with clear role definitions and preparatory training for mentors are recommended to improve support quality. For clinical practice settings, regular supervision and monitoring are needed to safeguard students from over‐dependence on peers and to ensure that peer support complements, rather than replaces, hands‐on clinical learning opportunities.

**Patient or Public Contribution:**

No patient or public contribution.

## 1 Introduction

Clinical practice is central to nursing education, bridging theoretical knowledge and clinical application (Yang and Zang 2022). It lays the practical foundation for nursing students' transition to professional nursing, which directly influences students' professional competence and career identity (Jacobsen et al. 2022). However, heavy clinical workloads and complex patient conditions often prevent preceptors from providing sufficient guidance. Adding to these pressures, role transition frequently leads to negative emotions that hinder clinical adaptation and skill development (Jokelainen et al. 2011).

Against this backdrop, peer support has gained attention as an effective supplement to clinical teaching. This educational support model involves participants with similar backgrounds sharing experiences to achieve learning and development goals collectively (Dennis 2003). Existing research confirms its ability to alleviate teaching pressures and enhance students' practical skills (Christiansen and Bell 2010). However, current studies remain fragmented and lack systematic integration, and the specific subjective perceptions and actual experiences of nursing students in peer support remain insufficiently understood.

Existing literature has explored the effectiveness of peer support across diverse clinical settings and student populations. Cuesta‐Martínez et al. (2024) found that Spanish nursing students participating in peer support programmes gained a sense of companionship, improved interpersonal skills and enhanced self‐confidence. Conversely, Ädel et al. (2021) noted that peer support can both promote health and pose obstacles to healthy development. However, globally, research on peer support among nursing students remains largely exploratory, with few qualitative studies examining their authentic experiences within these practices (Wu et al. 2023). By contrast, peer support research in nursing has long focused on disease‐specific areas like breast cancer, while studies targeting nursing interns remain scarce.

Therefore, this study employs a qualitative meta‐synthesis methodology to systematically retrieve and integrate relevant qualitative research up to 2024. It distills core findings to comprehensively understand the benefits, pressures and challenges students encounter when participating in peer support, clarifying their subjective needs. This provides a scientific reference for designing, optimising and implementing peer support programmes, thereby refining the peer support system in nursing education, alleviating students' practical pressures and enhancing the quality of clinical learning and practice.

## 2 Methods

Ethical approval was not required for this paper. The protocol for this study has been registered in PROSPERO, registration number: CRD42025649462.

### 2.1 Research Design and Methodological Framework

This study employs a qualitative meta‐synthesis design. It follows the Joanna Briggs Institute (JBI) ‘Qualitative Research Meta‐Synthesis Framework’, which provides standardised operational procedures for literature screening, data extraction and other stages, making it suitable for nursing and healthcare research. This framework ensures both the rigour and transparency of the synthesis process while preserving the contextual characteristics of qualitative data.

### 2.2 Data Sources and Search Strategy

We systematically searched seven electronic databases, including the Cochrane Library, Embase, PubMed, Web of Science, CNKI, Wanfang and VIP, for qualitative studies on nursing students' participation in peer support experiences, with the retrieval deadline set for 11 September 2024. Search terms were divided into two groups: the peer‐related terms included peer group, peer support, peer education, peer influence, trained peers, peer mentor, peer counselling, peer leader, peer discuss and peer coach; the population terms were nursing students and nursing interns. No language restrictions were imposed. Additionally, we manually screened the reference lists of all included articles to retrieve potentially eligible studies. The detailed search strategies for each database are provided in Supporting Information File 1. We also conducted supplementary searches in CINAHL and ERIC using identical search strings, but no additional eligible literature was retrieved.

### 2.3 Literature Screening Process

Literature screening was conducted independently by two researchers, with a third researcher resolving discrepancies through consensus discussion. The screening process strictly adhered to predefined inclusion and exclusion criteria and employed the PRISMA 2020 flowchart to ensure transparency and reproducibility.

### 2.4 Inclusion and Exclusion Criteria

Studies were included if they met the following criteria: (1) study population: nursing students involved in peer support, including mentors and mentees; (2) phenomenon of interest: nursing students' perceptions and experiences of peer support programmes during their clinical internships; (3) study context: nursing students' participation in peer support during their internships in hospitals and (4) type of study: qualitative or mixed‐methods studies with a qualitative component. Studies were excluded if they were (1) duplicate publications, (2) full text not available or (3) studies where peer support was not obtained during a hospital clinical placement.

### 2.5 Data Extraction

#### 2.5.1 Development of Data Extraction Form

The research team developed a customised data extraction form based on the JBI Qualitative Meta‐Analysis Guidelines and the objectives of this study. This form was not directly adapted from any single study but was designed to capture core information from all eligible qualitative studies. It underwent iterative adjustments to add or remove items, ensuring adaptability to the heterogeneity of the included literature.

#### 2.5.2 Content of the Data Extraction Form

The final form encompassed the following key research dimensions: (1) basic study information (author, year, country and sample size); (2) qualitative research methods (study design and data collection); (3) core research focus (clinical practice setting, nursing student level) and (4) key qualitative findings (peer support models and core concepts extracted from the included studies). Study limitations were not included in the data extraction form but were considered during quality appraisal and are addressed in the Limitations section.

#### 2.5.3 Data Extraction Implementation

Two researchers independently extracted data using the finalised form from the full text of each included study. Discrepancies in data extraction were resolved through cross‐checking with the original studies and consensus discussions with a third researcher. All extracted data were compiled into a Microsoft Excel spreadsheet for subsequent integration and analysis.

### 2.6 Article Screening and Quality Assessment

#### 2.6.1 Quality Assessment Tools and Process

Two researchers independently conducted methodological quality assessments of full‐text studies using the JBI Critical Appraisal Checklist for Qualitative Research. This validated tool evaluates study rigour across 10 dimensions. Researchers rated each dimension as ‘Yes (Y)’, ‘No (N)’, ‘Unclear (U)’ or ‘Not Applicable (N/A)’ based on the original research report.

#### 2.6.2 Quality Grading Criteria

Studies were assigned to three quality grades (A, B and C) based on the total number of ‘Yes’ ratings. Assessment and grading discrepancies were resolved through consensus with a third researcher.

#### 2.6.3 Post‐Assessment Inclusion Decision

A sensitivity inclusion strategy was applied. No Grade C studies were identified; all 10 included studies were Grade A (*n* = 3) or Grade B (*n* = 7), with no major methodological flaws compromising study validity.

#### 2.6.4 Assessment Reliability

To ensure assessment reliability, two researchers first calibrated their understanding of the JBI checklist through pilot assessments of two included studies. Inter‐rater reliability exceeded 0.8 (Cohen's *κ*), indicating high consistency in the evaluation process.

### 2.7 Data Integration and Theme Development

Data synthesis followed the JBI qualitative thematic synthesis three‐step approach (data extraction, data coding/categorization and synthesis of themes), conducted entirely by the research team to ensure rigour and interpretive validity. The process focused on the original qualitative findings and participant quotes included in the studies, aiming to generate new, comprehensive insights. Specifically, the research team first read and extracted 35 qualitative findings from 10 studies; subsequently, two researchers independently coded the findings using inductive content analysis, resolving discrepancies through team consensus to consolidate the 35 findings into 8 mutually exclusive subcategories. Based on conceptual connections, these subcategories were grouped into two overarching themes: ‘Benefits of Peer Support Experiences’ and ‘Stresses and Challenges of Peer Support Experiences’. Finally, a third senior researcher validated the themes, refined labels, cross‐checked original findings and integrated participant quotations to enhance credibility.

## 3 Results

### 3.1 Literature Search Findings

**FIGURE 1:**
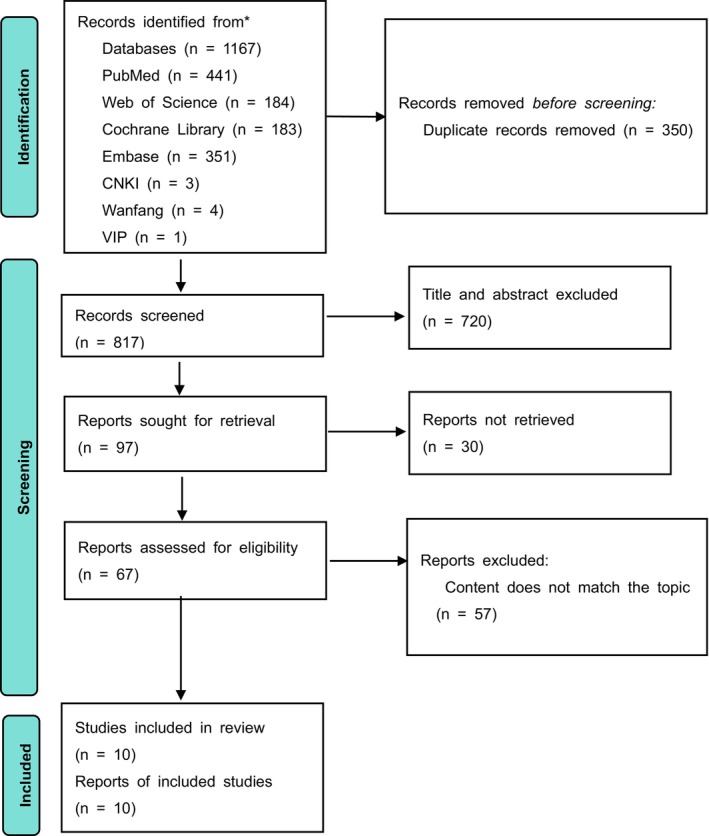
PRISMA 2020 literature screening and study inclusion flowchart.

A comprehensive literature search was conducted across seven electronic databases, supplemented by manual snowballing of reference lists. The literature screening process strictly adhered to the PRISMA 2020 statement guidelines, with the screening workflow illustrated in the PRISMA flow diagram (Figure 1). The initial database search yielded 1167 documents. After removing 350 duplicates, 817 documents remained. These underwent a title and abstract screening phase, resulting in the exclusion of 720 documents. Of the remaining 97 full texts, 30 were excluded due to incompleteness, leaving 67. A further 57 were excluded: 21 for implementing peer support in non‐hospital clinical settings, 15 for lacking qualitative findings on peer support experiences and 21 for being non‐original studies. Ultimately, 10 qualitative studies were included in this meta‐synthesis for in‐depth integrated analysis.

### 3.2 Basic Characteristics and Quality Assessment of Included Studies

A total of 10 qualitative studies published between 2004 and 2024 were included, covering 7 countries and involving 382 nursing students. The included studies varied in research design, clinical practice settings, educational levels of nursing students and peer support models. Specific characteristics are detailed in Table 1. All studies underwent methodological quality assessment using the JBI Critical Appraisal Checklist for Qualitative Research, covering 10 key methodological dimensions (see Table 2). No studies received a Grade C rating; 3 studies were graded A and 7 studies graded B, indicating overall high quality of the included literature and reliable findings from the qualitative research.

**TABLE 1:** Characteristics of included studies.

Author/year	Country	Study design	Data collection	Sample size (*n*)	Clinical practice setting	Nursing student level	Peer support model
Cuesta‐Martínez et al. (2024)	Spain	Descriptive qualitative research	Focus groups	107	Multi‐specialty (internal medicine/surgery/paediatrics)	Undergraduate	Group peer support
Carey et al. (2018)	UK	Ethnographic research	Non‐participant observation + semi‐structured interviews	62	Paediatric nursing	Undergraduate	One‐on‐one peer mentoring
du Plessis (2004)	South Africa	Descriptive qualitative research	Focus groups	45	General surgical nursing	Vocational	Group peer support
Loke and Chow (2007)	China	Qualitative research	Focus groups + semi‐structured interviews	30	Multi‐specialty (obstetrics/gynaecology/neonatology)	Undergraduate	One‐on‐one peer mentoring
Ravanipour et al. (2015)	Iran	Qualitative research	Focus groups + semi‐structured interviews	28	General medical nursing	Undergraduate	One‐on‐one peer mentoring
Austria et al. (2013)	USA	Phenomenological research	Semi‐structured interviews	11	Cardiac nursing	Undergraduate/postgraduate mix	One‐on‐one peer mentoring
Harmer et al. (2011)	USA	Mixed‐methods study (qualitative component)	Semi‐structured interviews	32	Multi‐specialty (emergency/general medicine)	Undergraduate	Group peer support
Roberts (2008)	UK	Ethnographic research	Non‐participant observation	15	Paediatric nursing	Vocational	One‐on‐one peer mentoring
Sprengel and Job (2004)	USA	Mixed‐methods study (qualitative component)	Focus groups	30	General medical/surgical nursing	Undergraduate	Group peer support
Ädel et al. (2021)	Sweden	Descriptive qualitative research	Semi‐structured interviews	22	Multi‐specialty (geriatrics/general medicine)	Undergraduate	One‐on‐one peer mentoring

**TABLE 2:** Methodological quality appraisal of the included studies.

Study	①	②	③	④	⑤	⑥	⑦	⑧	⑨	⑩	Quality level
Cuesta‐Martínez et al. (2024)	Y	Y	Y	Y	Y	N	N	Y	Y	Y	B
Carey et al. (2018)	Y	Y	Y	Y	Y	Y	Y	Y	Y	Y	A
du Plessis (2004)	U	Y	Y	Y	Y	N	N	Y	Y	Y	B
Loke and Chow (2007)	U	Y	Y	Y	Y	N	N	Y	U	Y	B
Ravanipour et al. (2015)	Y	Y	Y	Y	Y	N	N	Y	Y	Y	B
Austria et al. (2013)	Y	Y	Y	Y	Y	N	N	Y	Y	Y	B
Harmer et al. (2011)	U	Y	Y	Y	Y	N	N	Y	Y	Y	B
Roberts (2008)	Y	Y	Y	Y	Y	Y	Y	Y	Y	Y	A
Sprengel and Job (2004)	U	Y	Y	Y	Y	N	N	Y	U	Y	B
Ädel et al. (2021)	Y	Y	Y	Y	Y	Y	Y	Y	Y	Y	A

*Note:* Appraisal Checklist: ① Is there congruity between the stated philosophical perspective and the research methodology? ② Is there congruity between the research methodology and the research questions or objectives? ③ Is there congruity between the research methodology and the data collection methods? ④ Is there congruity between the research methodology and the representation and the data analysis? ⑤ Is there congruity between the research methodology and the interpretation of results? ⑥ Is there a statement locating the researcher culturally or theoretically? ⑦ Is the influence of the researcher on the research, and vice versa, addressed? ⑧ Are participants and their voices adequately represented? ⑨ Is the research ethical according to current criteria, and is there evidence of ethical approval by an appropriate body? ⑩ Do the conclusions drawn from the research report flow from the analysis or interpretation of the data? Appraisal result: Y; N; U and N/A.

Abbreviations: N = no, N/A = not applicable, U = unclear, Y = yes.

### 3.3 Synthesis Analysis

Through repeated reading, comprehension, inductive coding and interpretive analysis of the 10 included studies, the research team extracted 35 independent qualitative findings related to nursing students' experiences of peer support in clinical practice. Based on shared conceptual content, similar findings were categorised and integrated into eight new synthetic categories. These eight categories were then further synthesised into two overarching thematic findings based on positive/negative attributes. To enhance readability and facilitate rapid comprehension of core results, Table 3 summarises the eight categories and two overarching themes. Each category will be described in detail below.

**TABLE 3:** Core findings of meta‐synthesis: nursing students' peer support experiences in clinical practice.

Overarching theme	Integrated category	Core concept	Key supporting study (author/year)
Theme 1: Benefits of nursing students participation in peer support experiences (5 categories)	Increased self‐confidence and reduced anxiety	Peer support alleviates clinical practice anxiety for mentees; mentoring improves self‐efficacy for mentors	Cuesta‐Martínez et al. (2024), du Plessis (2004), Ravanipour et al. (2015)
	Sharing clinical experience and improving clinical skills	Peer mentors share practical clinical experience to help mentees link theory to practice and enhance clinical judgement/operative skills	Carey et al. (2018), Loke and Chow (2007), Roberts (2008)
	Learning together and modelling the power of example	Mutual learning between peers promotes learning motivation; competent peer mentors serve as role models for mentees	Cuesta‐Martínez et al. (2024), Loke and Chow (2007), du Plessis (2004)
	Gaining understanding, support and companionship	Peers with similar clinical experiences provide emotional empathy, companionship and a sense of security	Ädel et al. (2021), du Plessis (2004), Cuesta‐Martínez et al. (2024)
	Improved communication and interpersonal skills	Peer interaction enhances interpersonal communication ability; mentees learn patient communication skills from peer mentors	Cuesta‐Martínez et al. (2024), Carey et al. (2018), Loke and Chow (2007)
Theme 2: Stresses and challenges of nursing students participation in peer support experiences (3 categories)	Inadequate peer mentor competence	Variability in peer mentors' theoretical/clinical skills; some mentors lack the ability to answer mentees' questions	Ädel et al. (2021), Loke and Chow (2007)
	Different learning styles or personalities	Learning style mismatches hinder peer teaching efficiency; personality conflicts (e.g., dominant/controlling mentors) affect learning experience	Austria et al. (2013), Loke and Chow (2007)
	Reduced practice opportunities and increased dependency	Peer mentoring occupies mentees' hands‐on practice time; over‐reliance on peers weakens independent clinical practice ability	Ädel et al. (2021), Ravanipour et al. (2015), Austria et al. (2013)

#### 3.3.1 Theme 1: Benefits of Nursing Students' Participation in Peer Support Experiences

##### 3.3.1.1 Category 1: Increased Self‐Confidence and Reduced Anxiety

Peer support can increase self‐confidence for both nursing students and peer mentors. On the one hand, peer mentors initially doubted their own abilities and lacked confidence when they first took on mentoring roles, viewing the work as a challenging task, yet they built self‐confidence throughout the mentoring process (‘On the first day, I didn't believe that I would be able to work it out, and then I realised that I could help them, and that it was very good for my self‐confidence and my personal growth’ Cuesta‐Martínez et al. 2024). On the other hand, the clinical environment is unfamiliar to new nursing students on placement. They inevitably make errors and feel anxious, scared and nervous when facing the unfamiliar and complex hospital setting; peer mentors can help them adapt to clinical work and reduce their risk of clinical mistakes (‘My peer mentor helped me to adapt to the new environment and kept me calm’; du Plessis (2004). ‘I made mistakes that made me feel bad and nervous, but after they helped me, I don't make the same mistakes anymore’; Sprengel and Job 2004). Nursing students reported that when they encountered clinical difficulties, communicating with peers was much easier than consulting teachers, allowing them to feel more relaxed and less stressed, which further improved their self‐confidence (‘When the mentors were my peers, I was much less stressed because it was easier to talk about the problem with them than with the teachers, and naturally, there was more discussion and answering of questions, and confidence was increased’; Ravanipour et al. 2015. ‘Sometimes it is easier to ask for help from your peers than from your teacher because they are at the same level as you’; Austria et al. 2013).

##### 3.3.1.2 Category 2: Sharing Clinical Experience and Improving Clinical Skills

During clinical care work, peer mentors shared their clinical practice experiences to facilitate nursing students' understanding and learning (‘Babies are distressed and you can distract them with toys or other things to calm them down’ Carey et al. 2018). At the start of clinical placement, peer support assisted nursing students in connecting theoretical knowledge with clinical practice and enhancing their clinical judgement (‘When I first started my placement in obstetrics, I was unable to apply the theory in practice. With the help of my peers, I now encounter some clinical cases and can combine them with the theory in the textbook’; du Plessis 2004. ‘I am glad that my mentor taught me a lot and helped me in making better decisions in the clinical setting’; Loke and Chow 2007). Nursing students reported strengthening their clinical practice skills with the help of their peers (‘They helped me with practical exercises and helped me prepare for the practical exam’; Harmer et al. 2011. ‘They taught me what the icons on the monitors meant and informed me about what they did, which was important for me to acquire clinical skills’; Roberts 2008).

##### 3.3.1.3 Category 3: Learning Together and Modelling the Power of Example

Nursing students reported that they and their peer mentors could motivate and learn from one another (‘We prepared together, we studied together, we learned a lot’; Cuesta‐Martínez et al. 2024. ‘For example, in mother and baby care, which was unfamiliar to me initially, my peer mentor learned with me and I became more willing to ask questions and more motivated to learn’; Loke and Chow 2007). At the same time, the power of peer mentors as role models can motivate nursing students to learn and progress (‘I am glad that I can work and learn with competent mentors, which is very helpful for me to set an example of the power of role modelling’; du Plessis 2004).

##### 3.3.1.4 Category 4: Gaining Understanding, Support and Companionship

When experiencing difficulties in the clinical setting, nursing students reported that peers with similar experiences were more likely to understand them and be able to empathise (‘We were all able to talk to him; he understood us and he offered us solutions’; Cuesta‐Martínez et al. 2024. ‘I remember the first placement, I didn't know anything and having someone who knows and understands you really helps a lot’; du Plessis 2004). Nursing students felt that they did not feel alone even if their mentor did not do anything, but simply stayed with them and listened to what they had to say (‘I don't feel like I'm alone, at least I know someone who has been through the same thing and that relaxes me a little bit’; Ädel et al. 2021. ‘On my first day of placement I was nervous—it's a whole new experience and it's nice to have someone to go through the same thing with you’; Ädel et al. 2021).

##### 3.3.1.5 Category 5: Improved Communication and Interpersonal Skills

Since mentees and peer mentors are close in age, they can communicate naturally and freely (‘Talking to supervisors is nothing like talking to peers; you can call them by their first names, which makes communication much warmer’ Cuesta‐Martínez et al. 2024). Mentees are also willing to open up and share their inner feelings with peer mentors (‘When you step into a totally unfamiliar clinical environment and feel confused, you have a peer you can trust and confide in’; Cuesta‐Martínez et al. 2024). When interacting with patients, nursing students can learn practical communication skills from peer mentors (‘I learned how to communicate with people and how to actively understand patients' feelings’; Carey et al. 2018). They also master strategies to deal with uncooperative patients: ‘I used to avoid difficult patients, but with my peer mentor's guidance, I learned to provide clear explanations and adjust my way of speaking, making my interpersonal communication more flexible’; Loke and Chow 2007.

#### 3.3.2 Theme 2: Stresses and Challenges of Nursing Students' Participation in Peer Support Experiences

##### 3.3.2.1 Category 6: Inadequate Peer Mentor Competence

A small number of nursing students pointed out that peer support brought benefits, yet the professional competence of peer mentors varied greatly (‘Some peer mentors were not very competent or willing to help us’; Loke and Chow 2007. ‘Peer mentors didn't have enough knowledge to teach us and often couldn't answer our questions’; Ädel et al. 2021). These students hoped that peer mentors would be screened via strict selection procedures (‘Would like the process of selecting mentors to be more rigorous and thorough; this is very crucial’; Ädel et al. 2021).

##### 3.3.2.2 Category 7: Conflicting Learning Styles and Personality Differences

Some nursing students noted that inconsistent learning styles between the two parties can be a barrier to peer support (‘We have different learning styles and these can be difficult to work around’; Loke and Chow 2007) and that personality differences can be a hindrance to peer support (‘One of the two of us is very strong, so that person tends to talk more and control others more than the other, which will affect my learning’; Austria et al. 2013. ‘Although peer mentoring is useful, it is not good if one student is weak and the other peer does things for her’; Austria et al. 2013).

##### 3.3.2.3 Category 8: Reduced Hands‐On Practice Opportunities and Excessive Reliance on Peers

Nursing students perceived peer support as taking away from their own hands‐on practice time during clinical supervision, resulting in fewer opportunities for practice (‘Nursing practice is important, and of course, you can learn by observing your mentor, but I'd rather have less observing and more time to try to do it on my own’; Ädel et al. 2021. ‘I would rather have the opportunity to do nursing maneuvers for my patients instead of staying there and watching how my mentor does it’; Austria et al. 2013). In addition, they reported fewer opportunities and more dependence on them (‘If you can't find any opportunities to do these things independently, then you might be more dependent on your peers’; Ravanipour et al. 2015. ‘Although teamwork is useful, this method brings dependence’; Ravanipour et al. 2015).

## 4 Discussion

### 4.1 Validation of Findings Against Existing Literature

Existing research on peer support among nursing students demonstrates that qualitative and quantitative findings corroborate each other, collectively elucidating the positive effects of peer support, the challenges it faces in implementation and the corresponding practical implications and recommendations. Qualitative studies reveal that Spanish nursing students gain companionship, enhance interpersonal skills and build confidence through peer support, as reported by Cuesta‐Martínez et al. (2024), aligning with this study's theme of ‘Benefits of Peer Support Experiences’. Ädel et al. (2021) identified inadequate mentor capacity and reduced practical opportunities as primary barriers to peer support among Swedish nursing students, consistent with the theme of ‘Stressors and Challenges in Peer Support Experiences’. Loke and Chow (2007) reported dual outcomes of peer support among Chinese nursing students, aligning with the comprehensive findings of their meta‐analysis. In quantitative research, Christiansen and Bell (2010) quantified the positive impact of peer support on enhancing nursing students' clinical skills and reducing anxiety. Wu et al. (2023), although focused on cancer patients, provided corroborating evidence for confidence and interpersonal skill enhancement in nursing student peer support, and the study by Liu et al. (2023a) validated practical recommendations for optimising mentor selection and training mechanisms.

### 4.2 Raising Awareness of Peer Support Among Nursing Students to Optimise Its Positive Effects

The meta‐synthesis results indicate that peer support brings multiple positive outcomes for nursing students: boosting self‐confidence, alleviating clinical anxiety, advancing clinical skills, generating positive role‐modelling effects, offering emotional understanding and companionship and strengthening communication and interpersonal abilities.

Two interrelated mechanisms explain why peer support yields these benefits. First, peer mentors and mentees share identical learning stages and professional terminology, a concept termed cognitive congruence. This narrows the psychological gap commonly seen between students and clinical preceptors. When nursing students recognise that peer mentors have experienced the same clinical challenges, they feel secure enough to raise questions, acknowledge knowledge gaps and participate in learning proactively. Second, peer bonds create emotional congruence—an empathic connection rarely achievable within hierarchical teacher‐student relationships. This distinctive social support accounts for students' sense of being understood and their preference for sharing troubles with peers over clinical instructors.

Nevertheless, existing peer‐support literature mainly concentrates on patient groups with specific illnesses such as breast cancer, while peer support experiences of nursing students remain understudied and insufficiently recognised (Wu et al. 2023). Confronted with complicated clinical settings and unfamiliar nursing operations, numerous nursing students suffer from fear, anxiety, tension and other negative emotions. Clinical preceptors frequently lack adequate time for one‐on‐one guidance, which compromises clinical training quality and students' learning satisfaction (Jokelainen et al. 2011).

Peers are the most accessible partners for nursing students to communicate with, imitate and learn from. Similar age and learning backgrounds facilitate mutual discussion and joint progress. Unlike the hierarchical teacher‐student model, peer support has no power imbalance, rendering it more approachable and acceptable to students. Therefore, relevant departments should raise awareness of peer support among nursing students to fully exploit its advantages. Multiple online and offline approaches can be adopted to popularise its value, including offline face‐to‐face exchanges, online learning platforms and educational videos. These measures foster a mutual‐support atmosphere among nursing students, helping them advance collectively, refine clinical competencies and relieve clinical stress.

### 4.3 Analysing Pressures and Challenges in Peer Support to Optimise Support Programmes

This meta‐synthesis demonstrates that peer support delivers multiple merits, yet it is accompanied by prominent challenges including insufficient mentor competence, mismatched learning styles and personality conflicts, as well as excessive reliance on peers. The integrated findings further indicate that the effectiveness of peer support is determined by three contextual prerequisites. First, peer mentor competence is fundamental: peer support can exert positive effects only if mentors master solid clinical knowledge and basic teaching abilities. Second, interpersonal compatibility between mentors and mentees—consistent learning styles and matching personalities—profoundly influences learning outcomes. Third, reasonable teaching balance is essential: peer mentoring should serve as a supplement rather than a substitute for independent hands‐on practice. If these prerequisites cannot be satisfied, peer support may unintentionally limit students' independent learning opportunities instead of boosting their abilities.

Geyer et al. (2023) designed a peer support intervention for medical staff's mental health consisting of three stages: recruitment, training and formal implementation. Likewise, guided by social learning theory, Liu et al. (2023a) built a four‐stage peer support model for nursing students, covering supportive relationship establishment, peer behavioural observation, peer learning guidance and mutual feedback. Accordingly, we can refer to existing research to optimise the matching mechanism between mentors and mentees and formulate standardised peer support schemes.

First, peer mentors ought to be selected via rigorous screening procedures. Candidates must volunteer to participate and share similar learning and internship experiences with junior students. Meanwhile, their nursing theoretical knowledge and clinical operational proficiency will be assessed; only qualified candidates can serve as peer mentors to guarantee high‐quality guidance. In addition, the matching process should fully consider personality, learning style and value consistency between both parties to build harmonious supportive partnerships. These measures help consolidate stable peer support relationships and improve the quality of mentoring.

Second, systematic training should be provided for peer mentors to cultivate their communication capabilities. Mentors need to master active listening skills and appropriately share personal clinical experiences and lessons to encourage mentees and boost their self‐confidence.

Furthermore, reasonable time allocation must be emphasised during peer support practice. Peer tutoring time should not occupy most of the students' hands‐on practice time under team cooperation. This arrangement helps reduce students' over‐reliance on peers and motivates independent clinical operations. The core goal of peer support is to equip nursing students with the ability to complete clinical procedures independently, rather than making them rely entirely on mentors or peer groups. Mentors should guide mentees to think independently and provide more chances for them to demonstrate and practice clinical skills on their own to promote comprehensive growth.

Finally, diversified online and offline modes can be combined to enrich peer support approaches and sustain long‐term peer connections. Apart from offline face‐to‐face tutoring, in‐depth learning activities such as video teaching, scenario simulation and reflective journals can be applied. Virtual Reality (VR) and Augmented Reality (AR) technologies can also be introduced for operational training to further improve nursing students' clinical proficiency.

### 4.4 Promoting In‐Depth Emotional Communication Apart From Academic Learning to Build Sound Mutual Support Relationships

Nursing students are vulnerable to psychological distress due to their young age, unfamiliarity with complicated clinical settings, and the stressful role transition from student to clinical practitioner. Mental health constitutes a core component of their holistic well‐being and is closely associated with nursing students' professional identity formation and career planning (Hao et al. 2023). Existing evidence confirms that peer support can improve students' psychological resilience, strengthen their sense of professional gain, relieve stress and negative emotions and accelerate their adaptation to nursing roles (Liu et al. 2023b).

The findings of this meta‐synthesis further demonstrate that age‐matched peers are better able to provide empathy, attentive listening, companionship and practical coping strategies to ease the confusion and anxiety prevalent among nursing students during placement. Therefore, encouraging positive emotional communication between peers helps boost their psychological resilience, enables them to tackle internship challenges with a positive and healthy mindset, and ultimately promotes their smooth adaptation to clinical practice (Liu et al. 2023b).

To facilitate such emotional communication, interactive online groups can be set up through instant messaging software or social media platforms. Within these groups, nursing students can share clinical experiences, daily progress and inner emotional troubles. Sustained interaction in these groups cultivates mutual trust and solid supportive bonds, which further create a positive atmosphere for collaborative learning under peer support.

### 4.5 Implications for Nursing Practice and Nursing Education

This meta‐synthesis generates multiple operable implications for clinical nursing practice, nursing clinical education and hospital management.

#### 4.5.1 Mentor Selection and Standardised Training

Peer mentors should be screened via strict selection procedures that comprehensively evaluate their clinical theoretical knowledge, operational proficiency, communication skills, empathy and voluntary willingness to provide peer assistance. Before the peer support programme is launched, selected mentors need to participate in standardised systematic training, which includes role responsibilities, communication skills, active listening methods and constructive feedback skills. Fully trained mentors can provide high‐quality guidance and reduce adverse learning experiences for mentees.

#### 4.5.2 Role Definition and Regular Supervision

Clear responsibility boundaries between peer mentors and clinical preceptors should be defined to avoid role ambiguity. Peer mentors should act only as auxiliary supporters rather than substitutes for formal clinical instructors. Regular supervision meetings need to be arranged to track the quality of peer support, resolve emerging problems in a timely manner and guarantee that supporting activities match students' actual learning demands.

#### 4.5.3 Preventing Excessive Reliance on Peers

Programme managers and clinical instructors must guarantee that peer support serves as a supplement rather than a replacement for independent clinical practice. Students should be guided to take peers as learning resources while cultivating their own independent clinical thinking abilities. Once signs of over‐dependence appear, targeted intervention measures should be implemented immediately.

#### 4.5.4 Balancing Peer Tutoring and Independent Hands‐On Practice

The arrangement of peer support activities should reserve sufficient time for nursing students to carry out direct patient care and cultivate operational skills. The time spent on peer tutoring should not take up most of students' independent practice hours. Peer mentors and clinical preceptors should cooperate to balance peer‐assisted learning and autonomous skill training, so as to help students master core professional nursing competencies.

#### 4.5.5 Institutional Guarantee and Supporting Policy Formulation

Medical institutions and nursing colleges should jointly formulate standardised policies to incorporate peer support mechanisms into clinical internship schemes. Sufficient time, material resources and administrative support should be allocated to maintain the long‐term operation of peer support programmes. Routine evaluation and feedback collection are suggested to realise continuous quality improvement of the whole programme.

## 5 Limitations

This meta‐synthesis has several limitations. First, the authors' background in nursing education and qualitative research may have shaped our analytical interpretations and created a tendency to view peer support positively. To reduce such bias, we paid deliberate attention to all negative research outcomes, organised regular team discussions to review analytical interpretations, and reached consensus to resolve inconsistent coding results. Even so, interpretive subjectivity is an inherent limitation of qualitative interpretive synthesis.

Second, the included studies were conducted in different countries, various clinical environments and among nursing students at different educational levels, which may lead to heterogeneity bias. Subsequent research can further investigate nursing students' peer support experiences under diverse contextual conditions to optimise peer support implementation frameworks.

Third, among the 97 full‐text articles screened for eligibility, 30 papers failed to be retrieved. We made multiple retrieval attempts via institutional database subscriptions, academic platforms and direct correspondence with original authors. This may cause availability bias, since the inaccessible literature may contain unincorporated relevant findings. Nevertheless, the final sample of 10 studies covering seven countries forms a sufficiently diverse and information‐rich dataset to answer the core research question of this meta‐synthesis.

## 6 Conclusion

This meta‐synthesis reveals that peer support in clinical practice offers both benefits and challenges for nursing students. The benefits include enhanced confidence, emotional support and skill development, while the challenges relate to mentor competence, interpersonal dynamics and reduced hands‐on practice. A well‐structured peer support programme, with careful mentor selection, clear role definitions and ongoing supervision, is essential to maximise benefits and mitigate risks. Such programmes can reduce placement stress, improve clinical skills and ultimately enhance the quality of nursing education.

## Author Contributions

L.X. made substantial contributions to the conception and design of the study, data acquisition and manuscript preparation. L.X, Y.H. and W.X. screened the articles and performed quality assessment and data extraction. L.X., Y.H. and W.X. revised the manuscript for intellectual content. All authors have read and approved the final version of the manuscript.

## Funding

The authors have nothing to report.

## Ethics Statement

The authors have nothing to report.

## Conflicts of Interest

The authors declare no conflicts of interest.

## Supporting information


**Supporting Information: File 1.** Full search strategies for each database.

## Data Availability

The data that support the findings of this study are available from the corresponding author upon reasonable request.
