# Supplementary material for: Nursing Students' Experiences of Peer Support During Clinical Practice: A Qualitative Meta‐Synthesis
Source: Nurs Open. 2026 Jul 17;13(7):e70693. doi: 10.1002/nop2.70693 (PMC13376829; doi:10.1002/nop2.70693)
Supplement: Supplementary file 1 — Supporting Information: File 1. Full search strategies for each database. [file NOP2-13-e70693-s001.docx]

**Supplementary File 1: Full Search Strategies for Each Database**

All searches were performed on 11 September 2024. No date limits or language restrictions were applied.

**PubMed:**

("peer group"[MeSH] OR "peer support"[Title/Abstract] OR "peer education"[Title/Abstract] OR "peer influence"[Title/Abstract] OR "trained peers"[Title/Abstract] OR "peer mentor"[Title/Abstract] OR "peer counselling"[Title/Abstract] OR "peer leader"[Title/Abstract] OR "peer discuss"[Title/Abstract] OR "peer coach"[Title/Abstract]) AND ("nursing student"[MeSH] OR "nursing intern"[Title/Abstract])

**Embase:**

('peer group'/exp OR 'peer support':ab,ti OR 'peer education':ab,ti OR 'peer influence':ab,ti OR 'trained peers':ab,ti OR 'peer mentor':ab,ti OR 'peer counselling':ab,ti OR 'peer leader':ab,ti OR 'peer discuss':ab,ti OR 'peer coach':ab,ti) AND ('nursing student'/exp OR 'nursing intern':ab,ti)

**Cochrane Library:**

("peer group" OR "peer support" OR "peer education" OR "peer influence" OR "trained peers" OR "peer mentor" OR "peer counselling" OR "peer leader" OR "peer discuss" OR "peer coach") AND ("nursing student" OR "nursing intern")

**Web of Science:**

TS=("peer group" OR "peer support" OR "peer education" OR "peer influence" OR "trained peers" OR "peer mentor" OR "peer counselling" OR "peer leader" OR "peer discuss" OR "peer coach") AND TS=("nursing student" OR "nursing intern")

**CINAHL:**

("peer group" OR "peer support" OR "peer education" OR "peer influence" OR "trained peers" OR "peer mentor" OR "peer counselling" OR "peer leader" OR "peer discuss" OR "peer coach") AND ("nursing student" OR "nursing intern")

**ERIC:**

("peer group" OR "peer support" OR "peer education" OR "peer influence" OR "trained peers" OR "peer mentor" OR "peer counselling" OR "peer leader" OR "peer discuss" OR "peer coach") AND ("nursing student" OR "nursing intern")

**CNKI (中国知网):**

**Chinese search terms were used for Chinese databases (CNKI, Wanfang, and VIP).**

(SU='同伴支持' OR SU='同伴教育' OR SU='同伴指导' OR SU='同伴影响') AND (SU='护生' OR SU='实习护生' OR SU='护理实习生')

**Wanfang (万方):**

(主题:("同伴支持" OR "同伴教育" OR "同伴指导" OR "同伴影响")) AND (主题:("护生" OR "实习护生" OR "护理实习生"))

**VIP (维普):**

(T=("同伴支持" OR "同伴教育" OR "同伴指导" OR "同伴影响")) AND (T=("护生" OR "实习护生" OR "护理实习生"))
